# Moderate-Intensity Rotating Magnetic Fields Do Not Affect Bone Quality and Bone Remodeling in Hindlimb Suspended Rats

**DOI:** 10.1371/journal.pone.0102956

**Published:** 2014-07-21

**Authors:** Da Jing, Jing Cai, Yan Wu, Guanghao Shen, Mingming Zhai, Shichao Tong, Qiaoling Xu, Kangning Xie, Xiaoming Wu, Chi Tang, Xinmin Xu, Juan Liu, Wei Guo, Maogang Jiang, Erping Luo

**Affiliations:** 1 Department of Biomedical Engineering, Fourth Military Medical University, Xi’an, China; 2 Department of Endocrinology, Xijing hospital, Fourth Military Medical University, Xi’an, China; 3 Institute of Orthopaedics, Xijing hospital, Fourth Military Medical University, Xi’an, China; 4 Department of Nursing, Fourth Military Medical University, Xi’an, China; 5 Department of Medical Engineering, PLA No. 323 Hospital, Xi’an, China; Sudbury Regional Hospital, Canada

## Abstract

Abundant evidence has substantiated the positive effects of pulsed electromagnetic fields (PEMF) and static magnetic fields (SMF) on inhibiting osteopenia and promoting fracture healing. However, the osteogenic potential of rotating magnetic fields (RMF), another common electromagnetic application modality, remains poorly characterized thus far, although numerous commercial RMF treatment devices have been available on the market. Herein the impacts of RMF on osteoporotic bone microarchitecture, bone strength and bone metabolism were systematically investigated in hindlimb-unloaded (HU) rats. Thirty two 3-month-old male Sprague-Dawley rats were randomly assigned to the Control (*n* = 10), HU (*n* = 10) and HU with RMF exposure (HU+RMF, *n* = 12) groups. Rats in the HU+RMF group were subjected to daily 2-hour exposure to moderate-intensity RMF (ranging from 0.60 T to 0.38 T) at 7 Hz for 4 weeks. HU caused significant decreases in body mass and soleus muscle mass of rats, which were not obviously altered by RMF. Three-point bending test showed that the mechanical properties of femurs in HU rats, including maximum load, stiffness, energy absorption and elastic modulus were not markedly affected by RMF. µCT analysis demonstrated that 4-week RMF did not significantly prevent HU-induced deterioration of femoral trabecular and cortical bone microarchitecture. Serum biochemical analysis showed that RMF did not significantly change HU-induced decrease in serum bone formation markers and increase in bone resorption markers. Bone histomorphometric analysis further confirmed that RMF showed no impacts on bone remodeling in HU rats, as evidenced by unchanged mineral apposition rate, bone formation rate, osteoblast numbers and osteoclast numbers in cancellous bone. Together, our findings reveal that RMF do not significantly affect bone microstructure, bone mechanical strength and bone remodeling in HU-induced disuse osteoporotic rats. Our study indicates potentially obvious waveform-dependent effects of electromagnetic fields-stimulated osteogenesis, suggesting that RMF, at least in the present form, might not be an optimal modality for inhibiting disuse osteopenia/osteoporosis.

## Introduction

Osteoporosis, a progressive ‘silent bone disease’ caused by age, disuse or disease, is characterized by loss of bone mass and deterioration of bone microarchitecture, resulting in pain and deformity and increased risk of bone fracture [1], [2]. Bone loss due to the removal of weight-bearing physical activities, which occurs during therapeutic bed rest, limb immobilization and spaceflight, has become a non-negligible health concern in clinics and space medicine. Mechanical unloading induces negative skeletal calcium homeostasis, uncoupling of osteoclast and osteoblast activities, and resultant bone mineral loss [3]. Studies have shown that astronauts experienced loss of bone mineral density (BMD) with approximately 1.5% per month during spaceflight, equaling to that for postmenopausal women in 1 year [4], [5], [6]. It has been proved that individuals subjected to long-term bed rest or immobilization exhibited dramatic bone mass loss, deterioration of cancellous and cortical bone microarchitecture, and increased risk of falls and bone fracture [7], [8]. In view of the side effects or high cost of anti-osteoporosis drugs (e.g., bisphosphonates, calcitonin and hormones) [9], [10], [11], safe and noninvasive biophysical stimuli for the prevention and treatment of disuse osteoporosis might be more promising in clinical application, and especially favorable for the use of spaceflight.

Our growing understanding of the intricate piezoelectric and electromagnetic properties of bone tissues raised the possibility that exogenous electric or magnetic stimulation might regulate the activities and functions of bone cells. Since the 1970s when Bassett et al. for the first time promoted fracture healing in clinics using pulsed electromagnetic fields (PEMF) [12], abundant evidence has substantiated that electromagnetic fields (EMF) therapy was capable of producing satisfying therapeutic effects in a diverse range of bone diseases in the past four decades [13], [14]. The EMF has been approved by the FDA as a safe noninvasive treatment method in 1979. Basically, the three most common EMF application modalities well documented thus far include PEMF, static magnetic fields (SMF) and rotating magnetic fields (RMF). A large body of evidence has shown that PEMF displayed strong osteogenic potential both experimentally and clinically [15], [16], [17], [18], which was primarily associated with the induction of electric currents in tissues to initiate a battery of biological cascades. Several investigators also reported that SMF exposure was able to stimulate *in* *vivo* skeletal anabolic responses and increase BMD [19], [20]. Unlike PEMF, SMF induced no remarkable electrical potential in tissues and might directly affect bone cells through magnetic actions [20]. In recent years, several commercial therapeutic devices with RMF exposure are available on the market. Several previous studies have reported the beneficial effects of RMF on the musculoskeletal system [21], [22]. Zhang and colleagues found that 0.4 T RMF increase BMD and serum calcium and phosphatase (ALP) in ovariectomized (OVX) rats [21]. Pan et al. reported that 0.4 T RMF exposure mitigated hyperlipidaemia and steroid-induced necrosis of femoral head in rabbits [22]. However, to date the possible impacts of RMF on disuse-induced osteopenia/osteoporosis remain unknown. Thus, systemic assessment of the regulatory effects of RMF exposure on bone mass, bone microarchitecture, bone strength and bone metabolism in animal models of disuse-induced osteopenia is of great significance for the scientific application of RMF.

One of the best-recognized animal models to study disuse osteoporosis is the hindlimb unloading (HU) model via tail suspension [23], [24], which could induce decreased bone formation and increased bone resorption, and thus lead to the loss of bone mass and reduction of bone mechanical strength [25], [26]. Therefore, in the present investigation, the efficiency of RMF exposure on disuse-induced bone loss was systematically evaluated via analyses for serum biochemical, bone biomechanical, µCT and histomorphometric parameters in rats subjected to tail suspension.

## Materials and Methods

### Animals and experimental design

Thirty two mature 3-month-old male Sprague-Dawley rats (276.8±13.5 g, Vital River Laboratory Animal Technology, Beijing, China) were used in the present study. All procedures in the experiment were in strict accordance with the guiding principles of Institutional Animal Ethical Committee (IAEC), Committee for the Purpose of Control and Supervision of Experiments on Animals (CPCSEA), and the Guide for the Care and Use of Laboratory Animals published by the National Institutes of Health [NIH Publication.85–23]. The animal protocol was approved by the Institutional Animal Care and Use Committee of Fourth Military Medical University. All efforts were made to minimize the number of animals used. Animals were housed at 23±1°C temperature, 50%–60% relative humidity, 12:12 h light-dark cycle. Rats were randomly assigned to the Control (*n* = 10), HU (*n* = 10) and HU with RMF exposure (HU+RMF, *n* = 12) groups. The disuse of rat hindlimbs was induced by the tail-suspension technique according to the previously described protocol [27], [28]. Briefly, the tail after cleaned with 70% ethanol, was coated with a thin layer of liquid-like benzoin and resin dissolved in 99% ethanol. Then, a strip of adhesive tape was firmly attached laterally along the proximal portion of the tail and allowed thorough air dry, forming a loop close to the end of the tail. The adhesive tape was subsequently secured by three tape strips in its perpendicular direction. A plastic paperclip was employed to attach the loop of the surgical tape to a swivel hoop mounted at the top of a custom-designed plexiglass cage (length = 35 cm, width = 30 cm, height = 45 cm). The rat was maintained in an approximately 30° head-down-tilt position with its hindlimbs unloaded. Rats were caged individually and allowed free access to tap water and chow. The rats in the HU+RMF group were exposed to daily 2 h/day whole-body RMF for 4 weeks. Animals were intramuscularly injected with 25 mg/kg tetracycline (Sigma-Aldrich, Louis, MO, USA) at 14 and 13 days and 5 mg/kg calcein (Sigma-Aldrich) at 4 and 3 days before sacrifice, respectively. Rats were euthanatized with an overdose of chloral hydrate at the end of 4-week experiment. Serum samples were obtained via abdominal aorta puncture, centrifuged for 20 min and stored at –70°C for biochemical analysis. Bilateral femora were harvested, wrapped in saline-soaked gauze and stored at –70°C, which were used for mechanical testing and µCT analysis, respectively. Right tibiae were also harvested for bone histomorphometric analysis.

### RMF treatment

**Figure 1:**
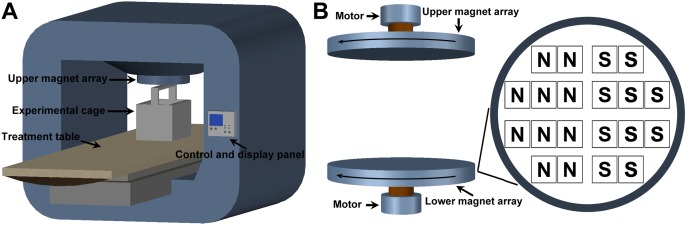
Schematic representation of the treatment device with RMF exposure used in the present study. (**A**) The therapeutic device mainly consists of a treatment table, two opposite anti-parallel arrays of NdFeB permanent magnets, and a signal display and control module. (**B**) Each magnet array comprises a total of 20 disc-shaped NdFeB magnets. The maximum magnetic flux density for each magnet is 400 mT. The right panel in (**B**) shows the network topology of the lower NdFeB permanent magnet array (**N** and **S** in the figure indicate the north pole and south pole of the magnet, respectively). The lower magnet array is rotated at 7 Hz driven by a high-power spinning motor, and thus driving the rotation of the upper magnet array. The rotation of both magnet arrays generates non-uniform RMF in the space between the arrays. The cage is placed coaxially with the upper and lower magnet arrays. The magnetic flux density distribution in the position of the cage region was determined to be 0.60–0.38 T.

As shown in **Fig. 1**, a commercial treatment system with RMF exposure (CRSMART-C, Chaoruishi Medical Supplies Co., Ltd, Zibo, China) was used in the present study. The therapeutic device mainly consisted of a treatment table, two opposite anti-parallel arrays of NdFeB permanent magnets, and a signal display and control module. Each magnet array comprised a total of 20 disc-shaped NdFeB magnets. The maximum magnetic flux density for each magnet was 400 mT. The network topology of the lower NdFeB permanent magnet array is shown in **Fig. 1B**. The lower magnet array was rotated at 7 Hz driven by a high-power spinning motor. The upper magnet array was also rotated accordingly at the same frequency driven by the upper motor. The rotation of both magnet arrays generated non-uniform RMF in the space between the arrays. The cage was placed coaxially with the upper and lower magnet arrays. A Gaussmeter (Model 455, Lake Shore Cryotronics, Westerville, OH, USA) with a transversal Hall Probe (HMFT-3E03-VF) was used to determine the spatial distribution of the magnetic field intensity. The determined magnetic flux density distribution in the position of the cage region ranged from 0.60 T to 0.38 T. The measured environmental background electromagnetic field was 0.5±0.02 Gs.

### Serum biochemical analysis

Commercial ELISA kits were employed for quantifying serum biochemical markers, including bone formation-associated osteocalcin (OC) and N-terminal propeptide of type 1 procollagen (P1NP), bone resorption-related tartrate-resistant acid phosphatase (TRAcP5b) and C-terminal cross-linked telopeptides of type I collagen (CTX-I) (Biomedical Technologies, Stoughton, MA, USA). ELISA kits were also used to determine the serum concentrations of two essential osteogenesis-associated cytokines, including prostaglandin E_2_ (PGE_2_) and transforming growth factor-β1 (TGF-β1) (JRDUN Biotechnology Co., Ltd., Shanghai, China). Assays were performed according to the manufacturers’ instructions.

### Biomechanical examination

The left femora after thawing in phosphate buffer solution (PBS) at room temperature were used for three-point bending test. The mechanical properties were evaluated at the femoral mid-diaphysis using a commercial mechanical testing system (*AGS*-*10* *kNG*, *Shimadzu*, Kyoto, Japan). The femur with its physiological curvature facing up was stabilized on a supporter with two fixed loading points with 20-mm distance. A stabilizing preload with 2 N was applied on the femoral medial surface using a steel cross-bar plate, which was oriented perpendicularly to the long axis of the sample and at the midpoint between the lower loading points. The bending load was applied at a constant displacement rate of 2 mm/min until fracture occurred. Then, the internal and external major axis and minor axis lengths of the femur at the fracture point were immediately measured using a vernier caliper. The following indices were determined from the load-deformation curve: maximum load (the maximum tensile load that the femur can sustain before failure), stiffness (slope of the linear part of the curve representing elastic deformation), and energy absorption (area under the load-deformation curve). Elastic modulus was calculated according to the formula: 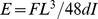, where *F* is the maximum load, *L* is the distance between supporting points, *d* is the displacement, *I* is the moment of inertia of the cross-section in relation to the horizontal axis.

### µCT analysis

The right femora of rats were scanned at a spatial resolution of 16 µm/slice using a high-resolution µCT system (GE healthcare, Madison, WI, USA). The femoral samples were placed in a 20-mm-diameter tube perpendicularly to the scanning axis with a total of 12-mm reconstruction height. After scanning, the 2-D image sequences were transferred to a workstation and 3-D images were reconstructed. For analyses of trabecular bone microarchitecture, a volume of interest (VOI) with 2.0-mm height was selected. The VOI started at a distance of 0.4 mm from the lowest end of the growth plate of the distal femur and extended to the proximal end with a distance of 2.0 mm, which excluded all the primary spongiosa and only contained the second spongiosa. The trabecular bone parameters, including trabecular BMD, trabecular number (Tb.N), trabecular thickness (Tb.Th), trabecular separation (Tb.Sp), bone volume per tissue volume (BV/TV), and structure model index (SMI) were automatically quantified using the MicroView program (GE healthcare, Madison, WI, USA). Moreover, the mid-diaphyseal cortical bone was manually traced by another VOI. The cortical bone parameters, including cortical thickness (Ct.Th) and cortical area (Ct.Ar) were also determined.

### Histology and histomorphometry

Right tibiae were immediately cut longitudinally into two pieces along the sagittal plane after animal dissection. One piece was fixed in 4% paraformaldehyde (PFA), decalcified in 10% ethylenediaminetetraacetic acid (EDTA), and embedded in paraffin. Five-µm-thick sections were stained with toluidine blue to visualize osteoblasts, and stained with tartrate resistant acid phosphatase (TRAP) to label osteoclasts. Static bone histomorphometric parameters, including osteoblast numbers per millimeter of trabecular bone surface (N.Ob/BS) and osteoclast numbers per millimeter of trabecular bone surface (N.Oc/BS) were quantified. The other piece was fixed in 80% ethanol for 24 h, and then embedded in methylmethacrylate. Eighty-µm-thick unstained sections were imaged with fluorescence microscope (LEICA DM LA, Leica Microsystems, Heidelberg, Germany) to observe and calculate the distance between the tetracycline and calcein labels divided by the labeling intervals of 10 days. Then, the dynamic bone histomorphometric parameters were quantified, including mineral apposition rate (MAR) and bone formation rate per bone surface (BFR/BS).

### Statistical analysis

All data presented in this study were expressed as the mean ± standard deviation (S.D.). Statistical analyses were performed using SPSS version 13.0 for Windows software (SPSS, Chicago, IL, USA). One-way analysis of variance (ANOVA) was employed for evaluating the existence of differences among the three groups and once a significant difference was detected, Bonferroni’s post hoc analysis was used to determine the significance between every two groups. The significance level was set at 0.05.

## Results

### Effects of RMF on body mass and soleus muscle mass in HU rats

The results of body mass and soleus muscle mass of rats before and after RMF exposure are shown in **Table 1**. No significant difference in body mass was observed between the Control, HU and HU+RMF groups before RMF exposure (*P*>0.05). HU for 4 weeks induced dramatic loss in body mass (*P*<0.01), soleus muscle mass (*P*<0.01) and soleus muscle mass normalized with body mass (*P*<0.01) as compared with the Control group; nevertheless, 4-week RMF exposure did not significantly alter the body mass, soleus muscle mass, or normalized soleus muscle mass in HU rats (*P*>0.05).

**Table 1:** Comparisons of body mass and soleus muscle mass of rats in the three experimental groups.

	Control (*n* = 10)	HU (*n* = 10)	HU+RMF (*n* = 12)
**Body Mass Day 0 (g)**	275.6±12.9	275.6±12.6	279.4±16.4
**Body Mass Day 29 (g)**	354.3±20.9	293.6±16.7*	291.4±14.4*
**Body Mass Change (g)**	78.6±14.5	18.0±12.5*	12.0±17.6*
**Soleus Mass (mg)**	174.9±15.7	79.0±13.3*	89.8±13.5*
**Soleus Mass/Body Mass (µg/g)**	494.3±42.7	270.4±51.1*	308.6±48.3*

Values are expressed as mean ± S.D.

*Significant difference from Control group with *P*<0.05.

### Effects of RMF on serum biochemical indices in HU rats

**Figure 2:**
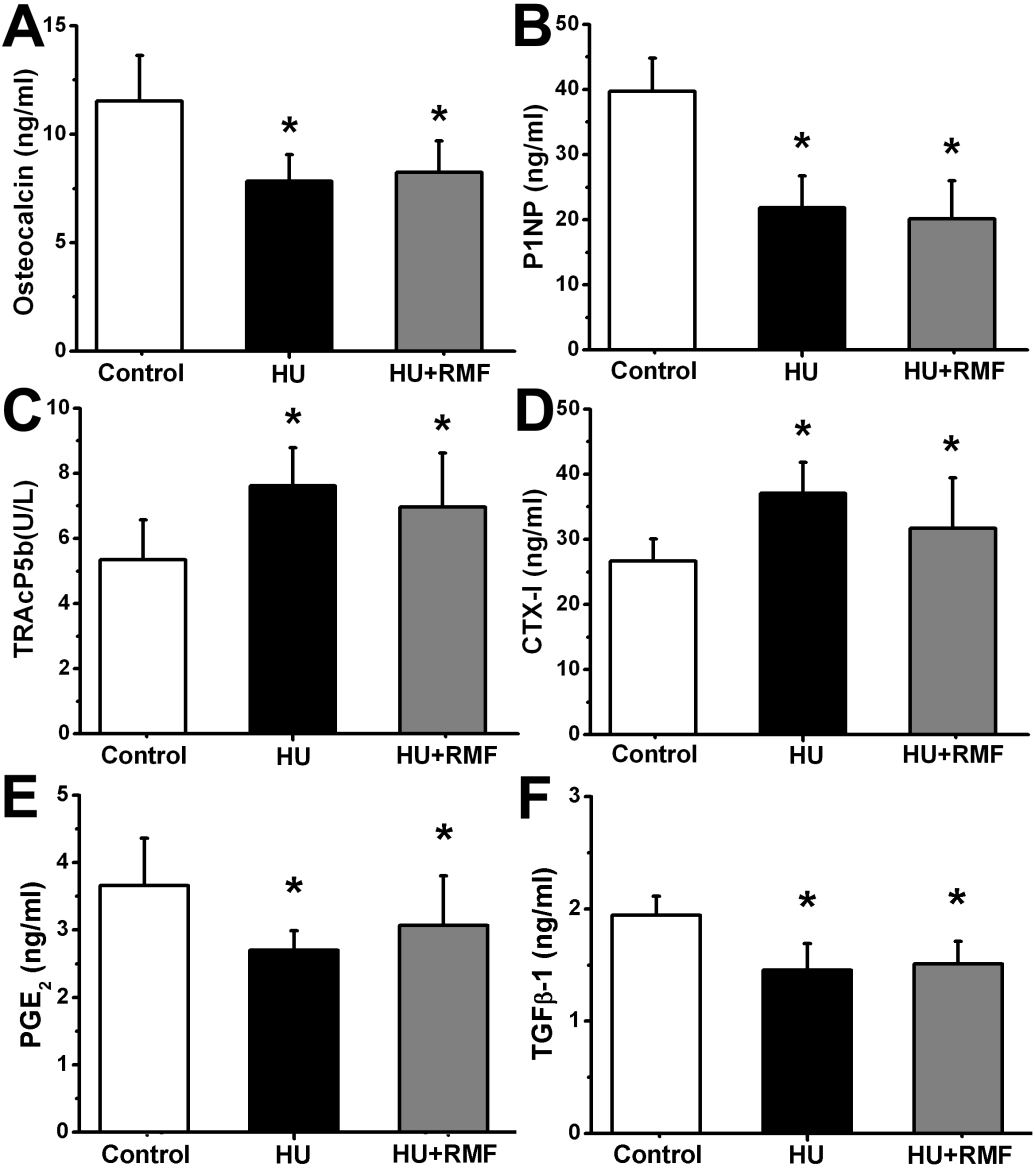
Effects of 4-week RMF exposure on serum biochemical indices (bone turnover markers and osteogenesis-associated cytokines) in HU rats, including bone formation markers (A) serum osteocalcin (OC) and (B) serum N-terminal propeptide of type 1 procollagen (P1NP), bone resorption markers (C) serum tartrate-resistant acid phosphatase (TRAcP5b) and (D) serum C-terminal cross-linked telopeptides of type I collagen (CTX-I), (E) prostaglandin E_2_ (PGE_2_), and (F) transforming growth factor-β1 (TGF-β1). Control, the control group (*n* = 10); HU, the hindlimb unloading group (*n* = 10); HU+RMF, the hindlimb unloading with RMF exposure group (*n* = 12). Values are all expressed as mean ± S.D. *Significant difference from the Control group with *P*<0.05.

As shown in **Fig. 2**, bone formation markers, including serum OC and P1NP in the HU group were significantly lower than those in the Control group (*P*<0.01). Serum TRAcP5 b and CTX-I, two serum markers for bone resorption, were remarkably higher in the HU group than those in the Control group (*P*<0.01). However, no significant difference in serum OC, P1NP, TRAcP5 b or CTX-I levels was found between the HU and HU+RMF groups (*P*>0.05). Moreover, serum levels of PGE_2_ and TGF-β1 in the HU group were lower than those in the Control group (*P*<0.05), whereas RMF did not significantly increase serum PGE_2_ or TGF-β1 concentrations (*P*>0.05).

### Effects of RMF on the biomechanical properties of bone in HU rats

**Figure 3:**
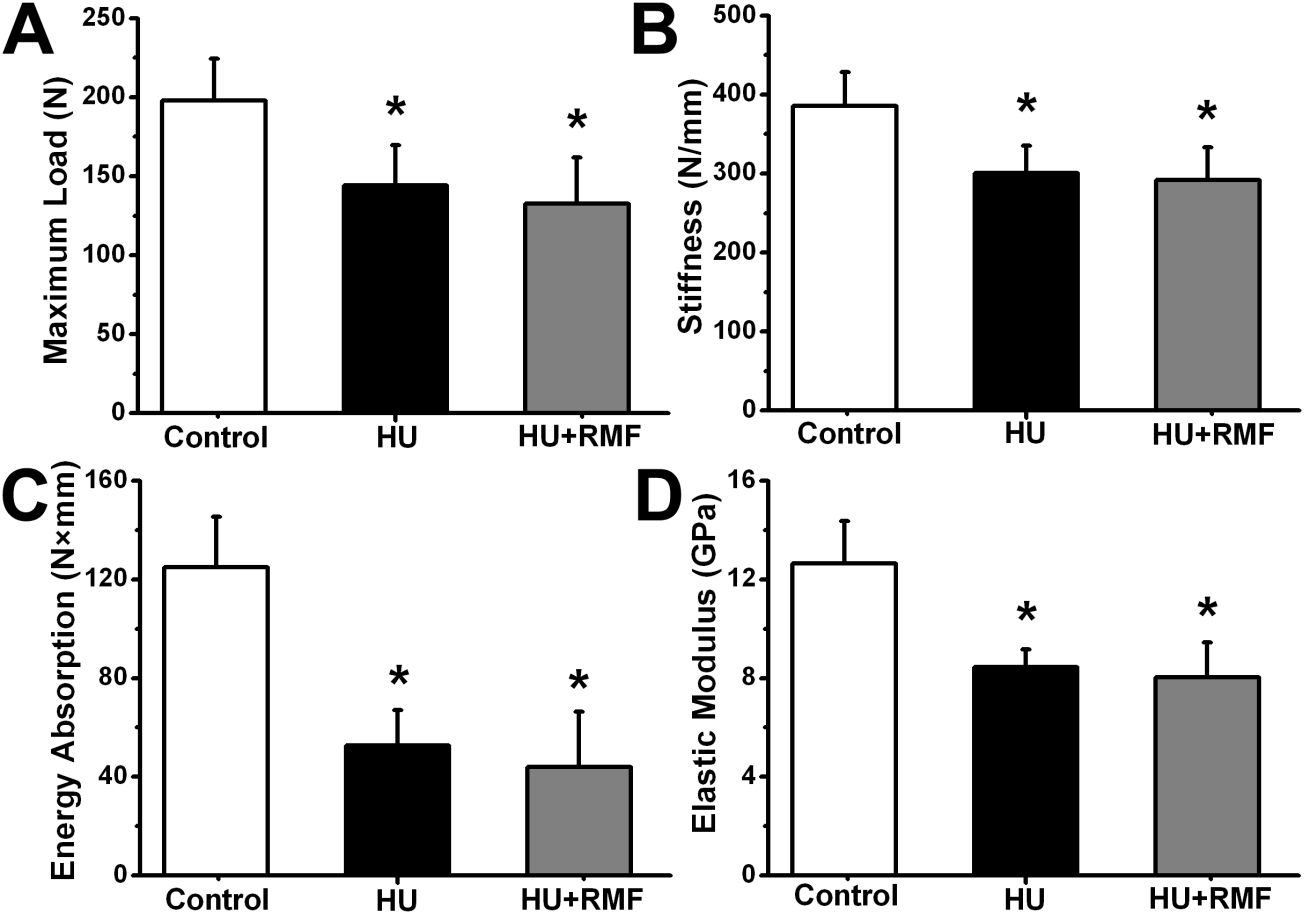
Effects of 4-week RMF exposure on femoral biomechanical parameters in HU rats, including (A) maximum load (B) stiffness (C) energy absorption and (D) elastic modulus. Control, the control group (*n* = 10); HU, the hindlimb unloading group (*n* = 10); HU+RMF, the hindlimb unloading with RMF exposure group (*n* = 12). Values are all expressed as mean ± S.D. *Significant difference from the Control group with *P*<0.05.

The results of biomechanical testing of three-point bending are shown in **Fig. 3**. HU resulted in prominent decreases in the biomechanical properties of femora, including maximum load, stiffness, energy absorption and elastic modulus (*P*<0.01), whereas RMF exposure for 4 weeks exerted no significant impacts on maximum load, stiffness, energy absorption or elastic modulus in HU rats (*P*>0.05).

### Effects of RMF on bone microarchitecture in HU rats

**Figure 4:**
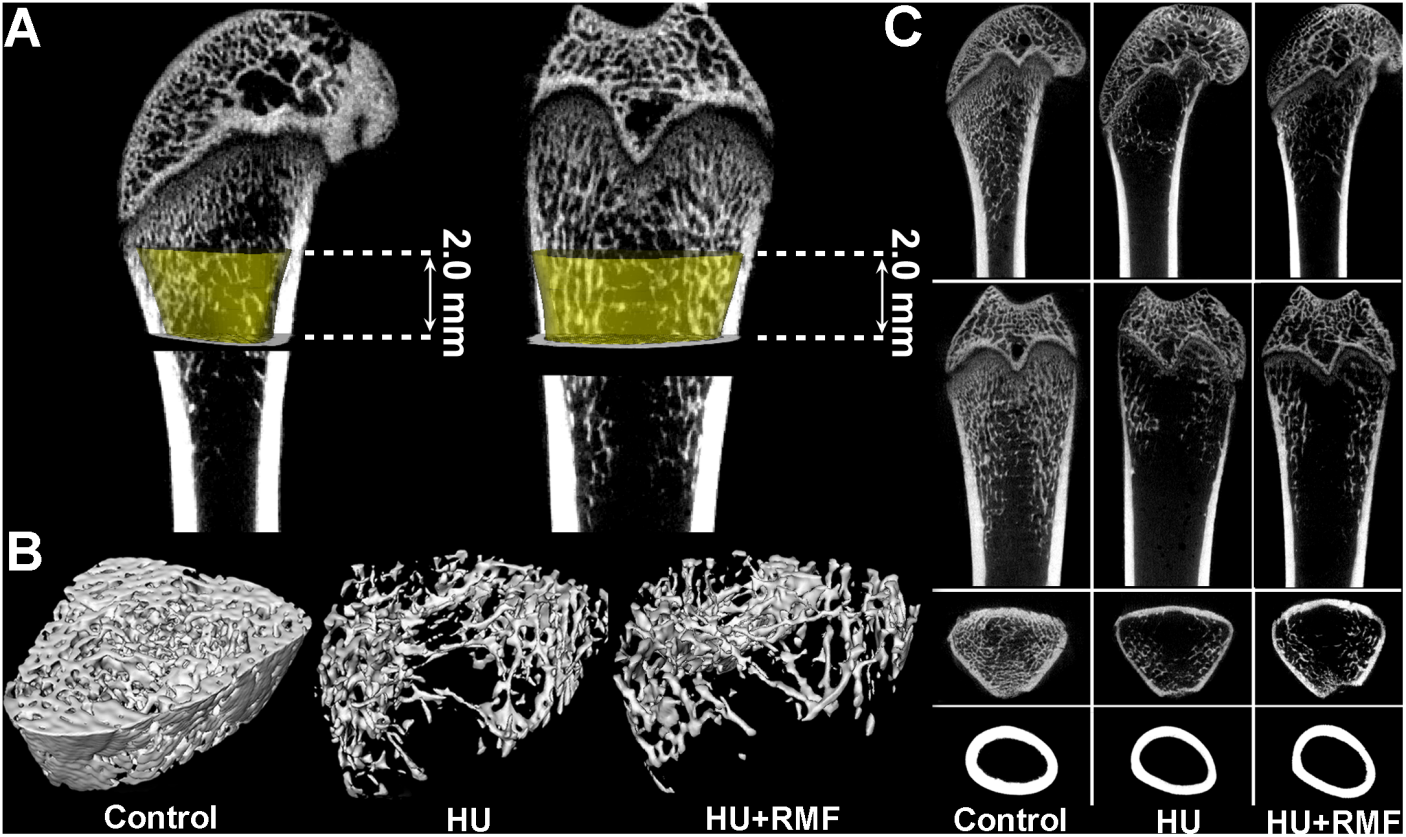
Effects of 4-week RMF exposure on trabecular bone microarchitecture in the distal femora and cortical bone thickness in the mid-diaphyseal femora. (**A**) The selected trabecular volume of interest (VOI) with yellow color in 2.0 mm height, which is represented with yellow color and only contains the secondary spongiosa. (**B**) 3-D µCT images of trabecular bone microarchitecture determined by the VOI. (**C**) 2-D µCT images of trabecular bone microarchitecture from the axial, coronal and sagittal plane observation in the distal femora, and cortical bone images in the femoral mid-diaphysis. The rat femur in the HU group exhibited significant decrease in the trabecular number, trabecular area and cortical thickness as compared with that in the Control group, whereas RMF exposure did not exhibit remarkable effects on trabecular bone microarchitecture and cortical bone thickness in HU rats.

**Figure 5:**
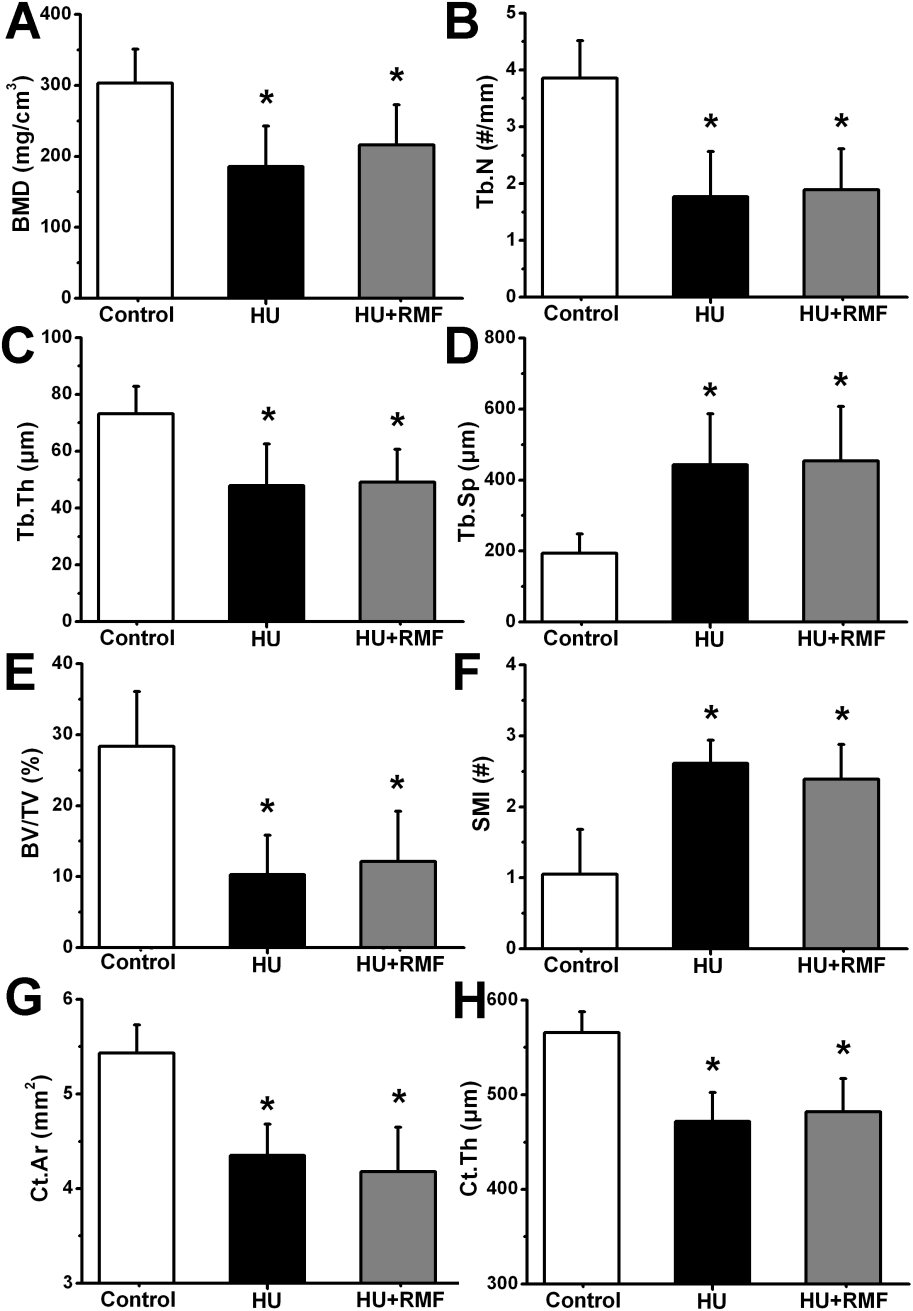
Effects of 4-week RMF exposure on µCT indices of femoral trabecular and cortical bone microstructure in HU rats, including (A) trabecular bone mineral density (BMD), (B) trabecular number (Tb.N), (C) trabecular thickness (Tb.Th), (D) trabecular separation (Tb.Sp), (E) bone volume per tissue volume (BV/TV), (F) structure model index (SMI), (G) cortical area (Ct.Ar) and (H) cortical thickness (Ct.Th). Control, the control group (*n* = 10); HU, the hindlimb unloading group (*n* = 10); HU+RMF, the hindlimb unloading with RMF exposure group (*n* = 12). Values are all expressed as mean ± S.D. *Significant difference from the Control group with *P*<0.05.

Representative 3-D and 2-D µCT images in the Control, HU and HU+RMF groups are shown in **Fig. 4**. The rat femur in the HU group exhibited notable reductions in the trabecular number, trabecular area and cortical thickness as compared with that in the Control group. RMF exposure did not exhibit remarkable effects on trabecular bone microarchitecture and cortical bone thickness in HU rats. The statistical results for the µCT analysis of trabecular and cortical bone structure in rat femora are shown in **Fig. 5**. Four-week skeletal disuse by HU caused significant decreases in trabecular BMD, Tb.N, Tb.Th and BV/TV (*P*<0.01), and increase in Tb.Sp and SMI (*P*<0.01). Moreover, HU induced significant deterioration in cortical bone structure of rat femora, as evidenced by decreased Ct.Ar and Ct.Th (*P*<0.01). However, no significant difference was observed in any trabecular or cortical bone parameter between the HU and HU+RMF groups, including BMD, Tb.N, Tb.Th, Tb.Sp, BV/TV, SMI, Ct.Ar or Ct.Th (*P*>0.05).

### Effects of RMF on bone remodeling in HU rats

**Figure 6:**
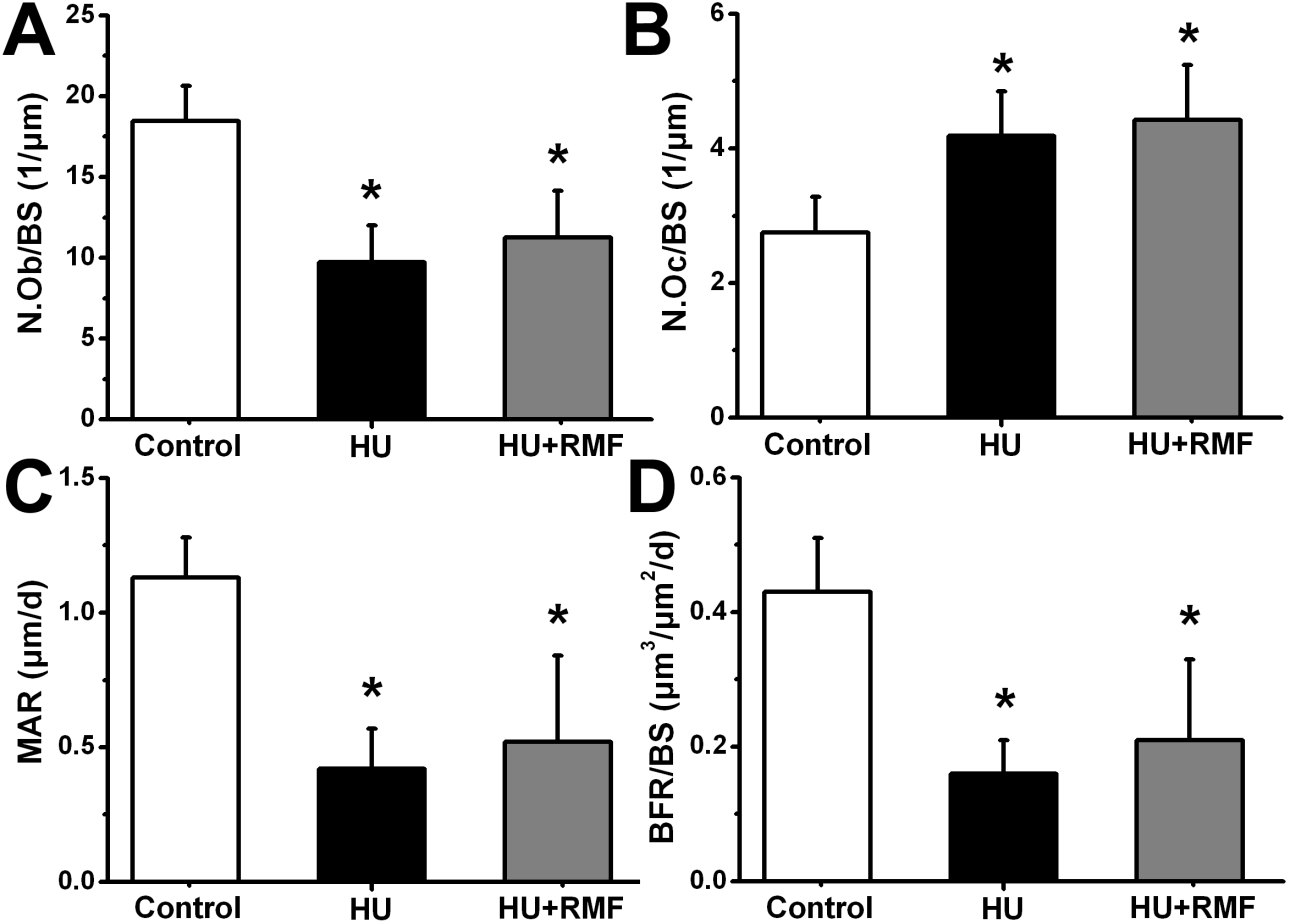
Effects of 4-week RMF exposure on tibial static and dynamic bone histomorphometric parameters in HU rats, including (A) osteoblast numbers per millimeter of trabecular bone surface (N.Ob/BS), (B) osteoclast numbers per millimeter of trabecular bone surface (N.Oc/BS), (C) mineral apposition rate (MAR) and (D) bone formation rate per bone surface (BFR/BS). Control, the control group (*n* = 10); HU, the hindlimb unloading group (*n* = 10); HU+RMF, the hindlimb unloading with RMF exposure group (*n* = 12). Values are all expressed as mean ± S.D. *Significant difference from the Control group with *P*<0.05.

As shown in **Fig. 6**, the HU rats exhibited significant decrease in N.Ob/BS (*P*<0.01) and increase in N.Oc/BS (*P*<0.01) comparing to those in the Control group; nevertheless, RMF exposure did not alter N.Ob/BS or N.Oc/BS in HU rats. Furthermore, HU for 4 weeks also led to significant decreases in MAR and BFR/BS (*P*<0.01). However, no significant difference in MAR or BFR/BS was found between the HU and HU+RMF groups (*P*>0.05).

### Effects of RMF on bone microstructure and bone turnover in normal rats

The descriptions for the methods for evaluating the effects of RMF on bone microstructure and bone turnover in normal rats are shown in File S1. As shown in **Fig. S1 in File S1**, RMF exposure did not significantly affect the parameters of bone microstructure in normal rats, including trabecular BMD, trabecular BV/TV or Cr.Ar (*P*>0.05). Moreover, no significant difference was observed in serum OC (the bone formation marker), and serum TRAcP5b (the bone resorption marker) between the Control and RMF groups (*P*>0.05).

## Discussion

Accumulating evidence has demonstrated the promotional effects of PEMF and SMF on osteogenesis both *in* *vivo and in* *vitro*, whereas few studies have reported the efficiency of RMF in the musculoskeletal system. A previous study has demonstrated that moderate-intensity RMF was able to increase BMD and regulate bone metabolism in OVX rats [21]. However, the impacts of RMF on disuse-induced osteopenia/osteoporosis have never been previously investigated. Therefore, in the present study, we systematically evaluated the effects of RMF on HU-induced bone loss in rats subjected to tail suspension. Our findings clearly demonstrated that 4-week RMF exposure did not obviously affect soleus muscle mass, bone mass, bone microarchitecture, bone mechanical strength or bone remodeling in HU rats.

In the present study, soleus muscle atrophy and deceased body mass in rats were induced by 4-week HU. Disturbed balance between protein synthesis and protein degradation are regarded to be the major mechanism of HU-induced muscle atrophy [29], although the exact signaling pathways and molecular mechanisms remain elusive. Previous investigation has demonstrated that pulsed electrical stimulation with 20 Hz has the potency to inhibit muscle atrophy by rescuing myonuclei and satellite cells [30]. It has also been shown that SMF exposure contributed significantly to the promotion of myogenic differentiation and myoblast alignment [31]. However, no obvious attenuation of soleus muscle atrophy in HU rats was observed after 4-week exposure to RMF in the present study.

The skeleton is a highly mechanoadaptive system, and insufficient mechanical stimuli to weight-bearing regions of the skeleton lead to bone mass loss [32], [33]. In line with previous studies [34], [35], HU resulted in marked deterioration in trabecular bone microarchitecture, as evidenced by decreased trabecular BMD, Tb.N, Tb.Th and BV/TV, and increased Tb.Sp. More importantly, increased trabecular SMI was also observed in HU rats, revealing a potentially dramatic reduction of trabeculae with plate-like structures [36]. Moreover, in accordance with previous findings [35], skeletal disuse by HU caused lower cortical bone thickness in the present study. Further observations by mechanical testing demonstrated that the skeletal extrinsic mechanical properties (maximum load, stiffness and energy absorption) and intrinsic mechanical properties (elastic modulus) were decreased in HU rats, implying the impaired mechanical integrity and declining capacity of fracture toughness [37]. However, RMF exposure did not obviously contribute to the improvement of trabecular bone microarchitecture, cortical bone thickness or bone mechanical strength.

To further evaluate whether RMF regulated osteoblastic and osteoclastic activities in HU rats, systemic analyses of serum biomarkers and bone histomorphometry for bone remodeling were performed. Similar with previous investigations [33], dramatically reduced bone formation was observed in HU rats, as evidenced by decreased serum markers (OC and P1NP) and histomorphometric parameters in trabecular bones (MAR, BFR/BS and N.Ob/BS). Our results also showed elevated serum TRAcP5b, serum CTX-I and N.Oc/BS in trabecular bones, revealing remarkably enhanced bone resorption induced by HU. However, we found no obvious regulatory effects of RMF on either serum markers or bone histomorphometric parameters for bone turnover, implying no direct impacts of RMF on skeletal anabolic or catabolic activities in HU rats. Moreover, many cytokines have proven to play essential roles in regulating the process of bone remodeling, such as PGE_2_ and TGF-β1. PGE_2_ is the most extensively produced prostanoid and has the capacity of stimulating bone formation and promoting fracture healing [38], [39]. Chang et al. showed that PEMF inhibited osteopenia in OVX rats and stimulated serum PGE_2_ secretions [40]. Significant evidence has demonstrated that TGF-β1 was able to regulate osteoblastic and osteoclastic functions [41]. More importantly, TGF-β1 has also proven to be an essential mediator for the coupling of dynamic bone resorption and bone formation [42], [43]. Our present study showed that serum PGE_2_ and TGF-β1 secretions were decreased in the absence of regular mechanical stimulation. However, 4-week RMF exposure did not significantly change the concentrations of serum PGE_2_ or TGF-β1.

The discovery of the skeletal piezoelectric effect by Fukada et al. in 1957 raised the possibility of the application of exogenous electrical stimulus on bone repair [44]. Subsequent studies have confirmed the osteogenic effects of electrical stimulation [45], [46]. Bassett et al. for the first time found that PEMF treatment, a more accessible and affordable non-contact modality, was able to dramatically accelerate fracture healing in patients [12]. Numerous studies have further proved that PEMF could promote potently osteogenesis and enhance bone mineralization both *in* *vivo* and *in* *vitro* [15], [16], [47]. Clinical investigations revealed that PEMF produced satisfying therapeutic effects on fresh and nonunion fractures and osteoporosis [14], [17]. It should be noted that the positive effects derived from PEMF stimulation revealed by Bassett et al. and other investigators were based on low-intensity and low-frequency non-thermal exposure levels, which were anticipated to primarily induce weak low-frequency electric current inside bone tissues. Moreover, these PEMF waveforms previously used were unidirectional single pulse or pulse burst. Several previous studies demonstrated that time-varying electromagnetic fields, e.g., sinusoidal wave, led to marked decreases of BMD and mechanical strength in rats [48]. Zhang et al. also demonstrated that sinusoidal EMF treatment decreased the osteoblasts proliferation and suppressed mineralized nodules formation, which exhibited opposite effects by unidirectional PEMF stimulation [47]. In the present study, moderate-intensity RMF we used could also induce spatial time-varying bidirectional electric fields in body tissues. However, different exposure waveforms, intensities and directionalities may contribute to the dramatically distinct effects between PEMF and RMF on the skeleton.

The beneficial effects of moderate-intensity and high-intensity SMF on osteogenesis have also been well documented thus far. Previous investigation has demonstrated that SMF promoted the differentiation and activation of osteoblasts *in* *vitro* [49]. It has been shown that SMF have the potency to enhance the local BMD in osteoporotic rats and accelerate fracture healing [19], [50], [51]. More interestingly, Kotani et al. found that high-intensity SMF exposure stimulated skeletal anabolic responses, and the orientation of bone formation and osteoblast growth was parallel to the magnetic field both *in* *vivo* and *in* *vitro* [20]. Thus, SMF may only induce a direct magnetic field to regulate osteoblast orientation, proliferation, differentiation, and bone formation, which shows distinct osteogenic mechanism with PEMF. However, unlike SMF with constant magnetic field direction, time-varying spatial magnetic fields generated by RMF we used in the present study might not facilitate the orientation of bone cells and their cytoskeletons, and thus might not facilitate stimulating bone formation in one particular direction. This might be one of the possible reasons for the minor impacts of RMF on bone quality and bone metabolism in HU rats in the present study.

Another interesting aspect for helping decipher the mechanism of RMF on the tissues is the quantification of the electromagnetic energy absorbed by the tissues and the heat effect because of the energy absorption. According to the previous description, the Specific Absorption Rate (SAR) of the tissue is calculated in the following equation [52]: SAR = σ·*E* ^2^/ρ, where σ is the conductivity of the tissue, ρ is the tissue mass, and *E* is the electric field intensity inside the tissue. The temperature alterations due to the energy absorption are able to be numerically quantified by analyzing the bioheat transfer equation based on the obtained SAR value [53]. A systematic numerical calculation based on finite element analysis will be performed in our following studies to obtain comprehensive understanding for the electromagnetic energy absorption in the tissues.

In conclusion, the present study demonstrated that exposure with moderate-intensity RMF at 7 Hz did not affect bone mass, bone microstructure, bone mechanical strength and bone remodeling in HU-induced osteoporotic rats, as evidenced by systemic evaluation for the serum biochemical, bone biomechanical, µCT and histomorphometric analyses. Although RMF stimulation may yield both a time-varying magnetic field and an electric current inside tissues, the RMF exposure was indeed not an optimal modality for regulating bone quality and bone remodeling, at least in its present form. It is regarded that treatment with EMF on various disorders probably exists “biological windows” of stimulus parameters. Thus, further investigations for the regulatory effects of high-intensity and low-intensity RMF on bone loss in HU rats are necessary to obtain a more comprehensive understanding for the osteogenic effects of RMF, which may be helpful for more scientific evaluation of RMF stimulation on osteopenia/osteoporosis in clinics.

## Supporting Information

File S1 **Combined file containing supporting materials and methods and Figure S1. Figure S1:** Effects of 4-week RMF exposure on femoral trabecular and cortical bone microarchitecture and serum markers for bone turnover in normal rats, including **(A)** trabecular bone mineral density (BMD), **(B)** trabecular bone volume per tissue volume (BV/TV), **(C)** cortical area (Ct.Ar), the bone formation marker **(D)** serum osteocalcin (OC), and bone resorption marker **(E)** serum tartrate-resistant acid phosphatase (TRAcP5b). Control, the control group; RMF, the RMF exposure group. Values are all expressed as mean ± S.D. (*n* = 8).
(DOCX)
