# Supplementary material for: Moderate-Intensity Rotating Magnetic Fields Do Not Affect Bone Quality and Bone Remodeling in Hindlimb Suspended Rats
Source: PLoS One. 2014 Jul 21;9(7):e102956. doi: 10.1371/journal.pone.0102956 (PMC4105536; doi:10.1371/journal.pone.0102956)
Supplement: File S1 — Combined file containing supporting materials and methods and Figure S1. Figure S1: Effects of 4-week RMF exposure on femoral trabecular and cortical bone microarchitecture and serum markers for bone turnover in normal rats, including (A) trabecular bone mineral density (BMD), (B) trabecular bone volume per tissue volume (BV/TV), (C) cortical area (Ct.Ar), the bone formation marker (D) serum osteocalcin (OC), and bone resorption marker (E) serum tartrate-resistant acid phosphatase (TRAcP5b). Control, the control group; RMF, the RMF exposure group. Values are all expressed as mean ± S.D. (n = 8). (DOCX) [file pone.0102956.s001.docx]

**SI Materials and Methods**

**Evaluating the effects of RMF on bone microstructure and bone turnover in** **normal rats**

To examine whether RMF exposure affected bone mircostructure and bone turnover in normal rats, sixteen 3-month-old male Sprague-Dawley rats (293.5 ± 15.2 g, Animal center of the Fourth Military Medical University, Xi'an, China) were used in the present study. Rats were randomly and equally assigned to the Control (*n*=8) and RMF exposure (RMF, *n*=8) groups. The rats in the RMF group were subjected to daily 2 h/day whole-body RMF exposure for 4 weeks. Rats were euthanatized with an overdose of chloral hydrate at the end of the 4-week experiment. Serum samples were obtained via abdominal aorta puncture, centrifuged for 20 min and stored at -70°C for biochemical analysis. The right femora were harvested, wrapped in saline-soaked gauze and stored at -70°C, which were used for µCT analysis. For the serum biochemical analysis, commercial ELISA kits were employed for quantifying serum biochemical markers, including bone formation-associated osteocalcin (OC) and bone resorption-related tartrate-resistant acid phosphatase (TRAcP5b). Assays were performed according to the protocols provided by the manufacturers. For µCT analysis, right femora were scanned using the µCT system (GE healthcare) at a spatial resolution of 16 μm/slice. After sample scanning and image reconstruction, a VOI with 2.0-mm height which started at a distance of 0.4 mm from the lowest end of the growth plate of the distal femur and extended to the proximal end with a distance of 2.0 mm was selected for the analysis of trabecular bone microarchitecture. The mid-diaphyseal cortical bone was manually traced by another VOI for the analysis of cortical bone structure. The critical parameters of bone microarchitecture, including trabecular BMD, BV/TV and Ct.Ar were quantified. **Figure S1**


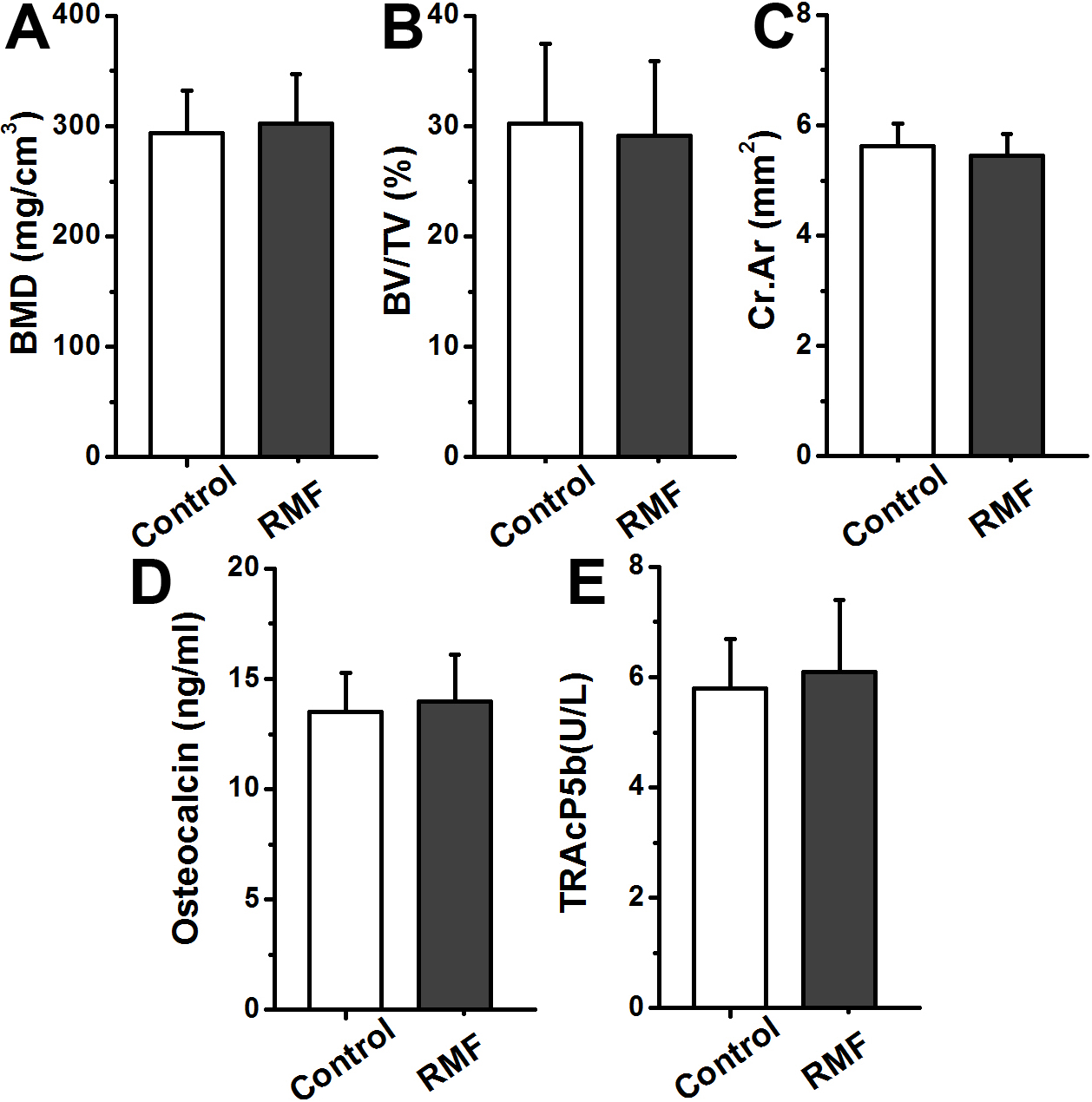
**Figure S1.** Effects of 4-week RMF exposure on femoral trabecular and cortical bone microarchitecture and serum markers for bone turnover in normal rats, including **(A)** trabecular bone mineral density (BMD), **(B)** trabecular bone volume per tissue volume (BV/TV), **(C)** cortical area (Ct.Ar), the bone formation marker **(D)** serum osteocalcin (OC), and bone resorption marker **(E)** serum tartrate-resistant acid phosphatase (TRAcP5b). Control, the control group; RMF, the RMF exposure group. Values are all expressed as mean ± S.D. (*n*=8).
